# Coexistence of zoophytophagous and phytozoophagous strategies linked to genotypic diet specialization in plant bug

**DOI:** 10.1371/journal.pone.0176369

**Published:** 2017-05-04

**Authors:** François Dumont, Eric Lucas, Denis Réale

**Affiliations:** Département des Sciences Biologiques, Université du Québec à Montréal, CP, Succ. Centre Ville, Montréal, QC, Canada; USDA Agricultural Research Service, UNITED STATES

## Abstract

Zoophytophagous predators can substitute zoophagy for phytophagy to complete their development and reproduction. In such predators, variation in feeding behaviour is observed both across and within populations. This may be caused by genetic variation in diet specialization, some genotypes specializing on plant resources, whereas others rely mostly on prey to meet their energy and nutriment requirements. We tested the hypothesis that genotypes specialize either on prey or plant resources in the zoophytophagous mullein bug *Campylomma verbasci*. In the laboratory, we reared 11 isogroup lines of the mullein bug and recorded feeding behaviour on two diets. The first diet was composed of two-spotted spider mites and leaves, and in the second we added pollen, a high-quality vegetal resource. Overall differences in zoophagy among isogroup lines remained consistent regardless of the presence or absence of pollen. While some lines were insensitive to changes in trophic resource composition others switched from prey to pollen feeding when the pollen was available, revealing a negative genetic correlation between the probabilities of feeding on both resources. A significant line by diet interaction in the proportion of time spent feeding on prey in presence or absence of pollen indicated a genetic basis for diet preference. In absence of the preferred resource, nymphs act as generalists, but exhibited individual diet specialisation when facing the choice between high-quality animal and vegetal resources. Results suggest that zoophytophagous predators can exhibit genetic variation in diet preference, which can generate important ecological and economic differences in natural or agricultural systems.

## Introduction

Zoophytophagous species can feed on either prey or plant resources to complete their development and reproduce [1, 2]. These species can be classified on a continuum ranging between phytozoophagy (i.e. herbivore that complements diet with prey) and zoophytophagous (i.e. predator that occasionally feeds on plant resources) based on their overall feeding habits [1]. The term 'zoophytophagous predators' is often used in studies on biological control to refer to predacious species than also feed on plant (and cause economic damages) [2]. However, because individuals within a species vary in their food resource preferences [3, 4], individuals can be classified on a zoophytophagous-phytozoophagous continuum when feeding habits are compared with conspecifics. Consuming and assimilating such a diversity of resources requires different morphological, physiological and behavioural adaptations [1], and could entail a trade-off between the efficiency of exploiting either prey or plant resources [3–5]. The diversity of zoophytophagous and phytozoophagous strategies in a population may result from gene by environment interactions [4].

Genetic differences in morphology (e.g. size, body shape), physiology (e.g. digestive enzymes), or behaviour (e.g. voracity) among individuals modulate their potential to change their diet in response to variation in ecological factors such as resource abundance, intra- and interspecific competition, or risk of predation [4, 6–8]. An individual’s ability to forage on different resources (e.g. prey *versus* plants) depends on the trade-offs between its ability to acquire, manipulate and digest each of these resources [4], and diet specialization should arise from such trade-offs [3, 9, 10]. Therefore, diet specialization in zoophytophagous predators would generate variation among individuals or genotypes in the responses to the availability of different resources. When different dietary resources are available to a species zoophytophagous genotypes would be expected to mostly feed on prey, whereas phytozoophagous genotypes would preferentially feed on plants. If one resource type is lacking, however, both genotypes would potentially feed on the remaining resource. Mixed diets should, thus, be more appropriate than homogeneous diets for detecting genetic differences in diet preferences and to reveal diet specialization in a population.

Using isogroup lines, we recently reported [11] genotypic variation in zoophagy in the zoophytophagous mullein bug *Campylomma verbasci* (Meyer) (Hemiptera: Miridae). In that study, bug isogroup lines differed considerably in the amount of prey [i.e. two-spotted spider mites *Tetranychus urticae* (Koch) (Acarina: Tetranychidae) and green peach aphids *Myzus persicae* (Sulzer) (Hemiptera: Aphididae)] they could kill over 24h. For instance, highly zoophagous lines killed an average of 42.75 mites by day, whereas moderately zoophagous lines only killed 11.13 mites by day. This difference had a high heritability (h^2^ = 0.54). Moreover, zoophagy on mites and aphids were positively correlated. Thus some isogroup lines were clearly more zoophagous than others. These results suggest the existence of potential diet specialization and of different foraging strategies in that species: some lines may consistently feed on animal resources and thus be considered zoophytophagous, whereas others may consume essentially vegetal resources and be considered phytozoophagous. However, the existence of these strategies has yet to be demonstrated in zoophytophagous predators.

The mullein bug is ubiquitous in Canadian apple and pear orchards [12]. Both adults and nymphs feed on prey, particularly the European red spider mite *Panonychus ulmi* (Koch) (Acarina: Tetranychidae) and two-spotted spider mites, as well as plant tissue (leaves, pollen, fruit) [12–14]. In the spring, after overwintering as eggs, young mullein bug nymphs emerged on apple tree (synchronized with both apple bloom and emergence of *P*. *ulmi*), and then complete five developmental stages before adulthood. Mullein nymphs feeding on fruitlets can cause economic damages, particularly to the Red Delicious cultivar [15]. The adults of the first generation either reproduce on apple trees or migrate to an herbaceous host such as the mullein plant (*Verbascum thapsus*). The summer generation nymphs, which are born from this first reproduction, may rely on different food resources based on their host. At that stage, apple tree pollen is no longer available and fruits are too large to be used as dietary resource. They, thus, feed mainly on prey and apple leaves. In contrast, on mullein plants, they feed mainly on pollen because mullein plants offer an extended flowering period, but have few mullein bug prey. Second-generation adults fly back to the orchard to reproduce and lay overwintering eggs in tinder apple branches.

In the present study, we tested for the effect of plant resource (pollen) availability on feeding behaviour and on the propensity to feed on animal resources (spider mites) using 11 isogroup lines of the zoophytophagous mullein bug. Our hypothesis was that genetic differences in diet specialization towards either a zoophytophagous or a phytozoophagous strategy affect the ability to switch from prey to plant resources in response to pollen availability. We predict that: 1) propensity to feed on prey and on pollen varies among lines. In a previous study, we observed significant among-line differences in zoophagy [11]. Such along-line differences should also be observed in the consumption of pollen. 2) When offered the choice between prey and pollen, individuals choose preferentially the resource that matches their foraging strategy (i.e. animal-based diet or plant-based diet). Therefore, we expect a negative correlation between the propensity to feed on either prey or pollen, and that the availability of pollen leads phytozoophagous lines to adopt diets with a low proportion of prey in contrast to the zoophytophagous lines.

## Methodology

### Mullein bug populations and isogroup lines

Mullein bug nymphs (*C*. *verbasci*) were reared in the biological control laboratory at Université du Québec à Montréal (Montréal, Québec). Founder individuals originated from both apple trees and mullein plants collected in different regions of Québec (i.e. Laurentians 45.526760°N/ -73.955322°E, Estrie 45.374509°N/ -71.920924°E, Montérégie 45.457841°N/ -73.033144°E, Québec 46.975416°N/ -70.952636°E, Montréal 45.513286°N/ -73.583486°E). During autumn in 2011 and 2012, apple tree cuttings were collected in orchards and stored in a refrigerated room to allow eggs to complete their diapause (1°C, 60% relative humidity). In February, each cutting was placed in an acrylic glass cage at 25°C, 60% RH and 16:8 [L:D] photoperiod until nymphs hatched (about 10 to 12 days). Nymphs were manually collected with a fine paintbrush and transferred to a 10 cm diameter petri dish containing fresh cut mullein, potato and soybean leaves inserted in agar gelatine to keep them moist. Mullein bug nymphs were supplied with green peach aphids *Myzus persicae* (Sulzer) (Hemiptera: Aphididae), two-spotted spider mites *Tetranychus urticae* (Koch) (Acarina: Tetranychidae) and pollen (biological flower pollen composed of about 15 g of protein in 100 g of pollen). We also used mullein bugs from a laboratory reared colony founded in 2007. Individuals from this rearing originated from orchards in the Laurentians (Québec). Both green peach aphids and two-spotted spider mites came from stocks previously maintained in our laboratory.

Two female and two male mullein bugs were used to establish each isogroup line (referred to as line thereafter). Founders of each line were randomly picked from the main population, which was composed of field-captured bugs and a stock population reared in the lab since 2007. Individuals were not matched based on their origin or on their foraging behaviour. Using two pairs of bugs at the beginning of each line reduced considerably the rate of extinction of a line, which suggests that most lines were founded by one of the two pairs. Lines were held in a 12 X 12 X 16 inch acrylic glass cage containing mullein (x 1), soybean (x 1) and potatoe (x 2) plants. Green peach aphids, two-spotted spider mites and pollen were provided *ad libitum*. Plants were replaced when necessary (about every 10 days for soybean and potatoes plants and 30 days for mullein plants). Lines were maintained in this system for up to 618 days after the foundation (i.e. about 16 generations). Foraging behaviour tests were run between the 2nd and the 15th generation, assuming a 40-day generation length (between 62 to 618 days after the foundation of lines). Individuals from different generations were tested for each line. The number of individuals tested within each generation (ranging from 0 to 9) depended on their availability.

Isogroup lines, or isofemale lines when only single males and females are used as a founder, are commonly used to estimate the genetic variance in a given trait within a studied population [16, 17]. Because of the strong founder effect and genetic drift occurring during the creation of an isogroup line, each line represents a genetic subsample of the original population, and after a certain number of generations within-line genetic variance is negligible. To minimize the proportion of environmental variance in the phenotypic variance, the lines are reared and individuals are tested under controlled conditions. It is, therefore, possible to estimate genetic variance of a trait by estimating the among-line variance for that trait [16].

### Tests on feeding behaviour

The feeding behaviour of 3^rd^ to 5^th^ instar mullein bug nymphs was observed in 10 cm petri dish that contained cuts of one fresh mullein leaf and one fresh soybean leaf inserted into agar gel. In the without-pollen treatment, 100 two-spotted spider mite nymphs and adults were manually transferred with a fine brush. Most spider mites remained on the soybean leaf where they foraged and laid their eggs. In the treatment with pollen, we added 5 mg of pollen to the mullein leaf.

Prior to the tests, we put mullein bug nymphs from each line individually in a Petri dish containing a standardized diet of mullein leaf on agar gel for 24 hours (this standard diet mainly provided water to the bug) and maintained in a growth chamber at 24°C, 60% RH and a [16L:8D] photoperiod. The day of the test we noted the developmental stage of each nymph and gently deposited it in Petri dishes containing a soybean leaf infested with two-spotted spider mites (both in treatments with and without pollen).

The behaviour of each nymph was observed under a dissecting microscope (10x) for 15 minutes. Tests were run between 10:00 am and 3:00 pm. We voice-recorded the behaviour of the individuals and noted the time and the type of animal resources (i.e. all forms of two-spotted spider mites) and vegetal resources (i.e. soybean and mullein leaf, pollen) the nymph consumed. During feeding, mullein bug nymphs bend the first and second labial segments of the rostrum to penetrate a food source, and inject a mixture of saliva and diluted matter [18]. Exploratory probing (e.g. deeper penetration of the stylet) tends to be of short duration [18]. Thus, when the maxillary stylet is inserted the food source, the bug is flushing out the contents of the food resource. We thus considered that a nymph had fed on a given resource when its maxillary stylet was inserted into the food item. From these observations, we measured the time spent consuming each type of resource (i.e. animal and vegetal). We could thus estimate the proportion of time feeding on a given prey (i.e. spider mite adults, nymphs or eggs) over the total time spent foraging on available resources. We also recorded the occurrence of feeding on prey and on pollen as binomial data (feeding or not feeding over a 15-minute interval). We ran tests on 155 mullein bug nymphs for a total of 41.5 hours of observations (i.e. 5–12 individuals per line per treatment). In 48 trials, nymphs did not consume any resource and the observations were discarded from the analysis; 107 tests remained in the analysis.

### Statistical analysis

All analyses were implemented using R [19] and the *lmer* function of the *lme4* library [20, 21].

We first tested for the effect of pollen availability on the probability of feeding on spider mites during a 15 min trial (each nymph was scored 1 if observed feeding on prey and 0 if not; n = 107). Data followed a binomial distribution and we thus used a generalized linear mixed-effect model (GLMM) for binomial data. We included treatment (with or without-pollen), developmental stage (N3 to N5) and generation (centered on the mean) as fixed effects. The random structure was selected comparing 11 models that varied in their random effect structure involving line ID, treatment and generation (see Table 1 for details). The model with the lowest Akaike Information Criterion (AIC) was selected as the best-fitted model [22, 23].

**Table 1:** Akaike Information Criterion (AIC) for the different random effect structure of a generalized linear mixed-model on mullein bug's probability to feed on prey and to feed on pollen, and the proportion of animal resources in their diet (107 individuals tested from 11 isogroup lines). The fixed structure of the model included generation (centered on the mean of each line), development stage and treatment (with or without pollen). Selected models, based on the lowest AIC, are in bold. The letter C in parenthesis beside a random slope means that the correlation between the random slope and the random intercept was implemented in the model. The indication "Corr" in the random slopes means that the correlation between the random slopes was implemented in the model.

Random effects		Akaike Information Criterion (AIC)		
Random slopes	Random intercept	Fed on prey	Fed on pollen	Proportion of animal resources
	line ID	**121.13**	**81.63**	9591.17
generation *(c)*	line ID	124.80	85.53	6911.55
generation		125.06	82.91	8937.37
generation	line ID	123.13	83.63	6915.97
diet *(c)* + generation	line ID	126.96		5215.03
diet + generation		126.96		5215.03
diet + generation	line ID	129.13		5217.03
diet + generation + corr	line ID	133.0		5202.75
*diet (c)* + *generation (c)* + corr	line ID	129.81		**5200.75**
diet		124.96		7344.42
diet *(c)*	line ID	124.96		7344.42

We tested for differences among the lines in the probability of feeding on pollen (referred to as phytophagy thereafter), by running a GLMM for binomial data. Developmental stage (N3 to N5) and generation (centered on the mean) were included as fixed effects. Four models were compared with or without line ID or the interaction between line ID and generation as two random effects. In these models, data from observations of individuals without pollen were discarded (n = 56). We then used our GLMM models to estimate the genetic correlations between the probability of feeding on prey and on pollen using best linear unbiased predictors (BLUPs).

We also analysed the proportion of time spent feeding on animal resources (referred to as zoophagy thereafter) over the total time spent feeding as a function of treatment (with or without-pollen), development stage and generation using two GLMMs for binomially distributed data (n = 107). We included line ID, treatment, generation and their interaction as random effects. The random structure was determined by comparing the AIC of 11 models that varied with random effects included (see Table 1). For the three models, we used a likelihood ratio test (LRT) to estimate the statistical significance of fixed effects (using the function *drop1* in R and the ML option) [20].

Mullein bugs were tested at different generations. Some variation among individuals of the same line (i.e. within-line variance) resulted from genetic drift [24]. The number of generations between the test and the foundation of the line (centered on each line's mean) was thus included as a covariate in the compared models. Models with and without a correlation among random effects were compared on the basis of their AIC. If the best-fit model included a correlation, it indicated significant differences among lines in the change in behaviour with generation, whereas a correlation between intercepts and slopes provided information on how the lines differ in their behaviour with time.

We estimated the upper limit of narrow heritability (h^2^) in each model based on the isogroup lines repeatability (R) of these behaviours (following [25]). The following equations was used for binomial GLMM models:

$$R=\frac{\sigma_{\alpha }^{2}}{\sigma_{\alpha }^{2}+\sigma_{\varepsilon }^{2}+\pi^{2/3}}$$

Where the residual variance $\sigma_{\varepsilon }^{2}$ is assumed to be 0 in normal binomial models and *π*^2/3^ represents the distribution-specific variance of a logit-link structure. Heritability (*h*^2^) on each treatment was measured independently when the random structure of the selected model included the variable treatment.

## Results

**Fig 1:**
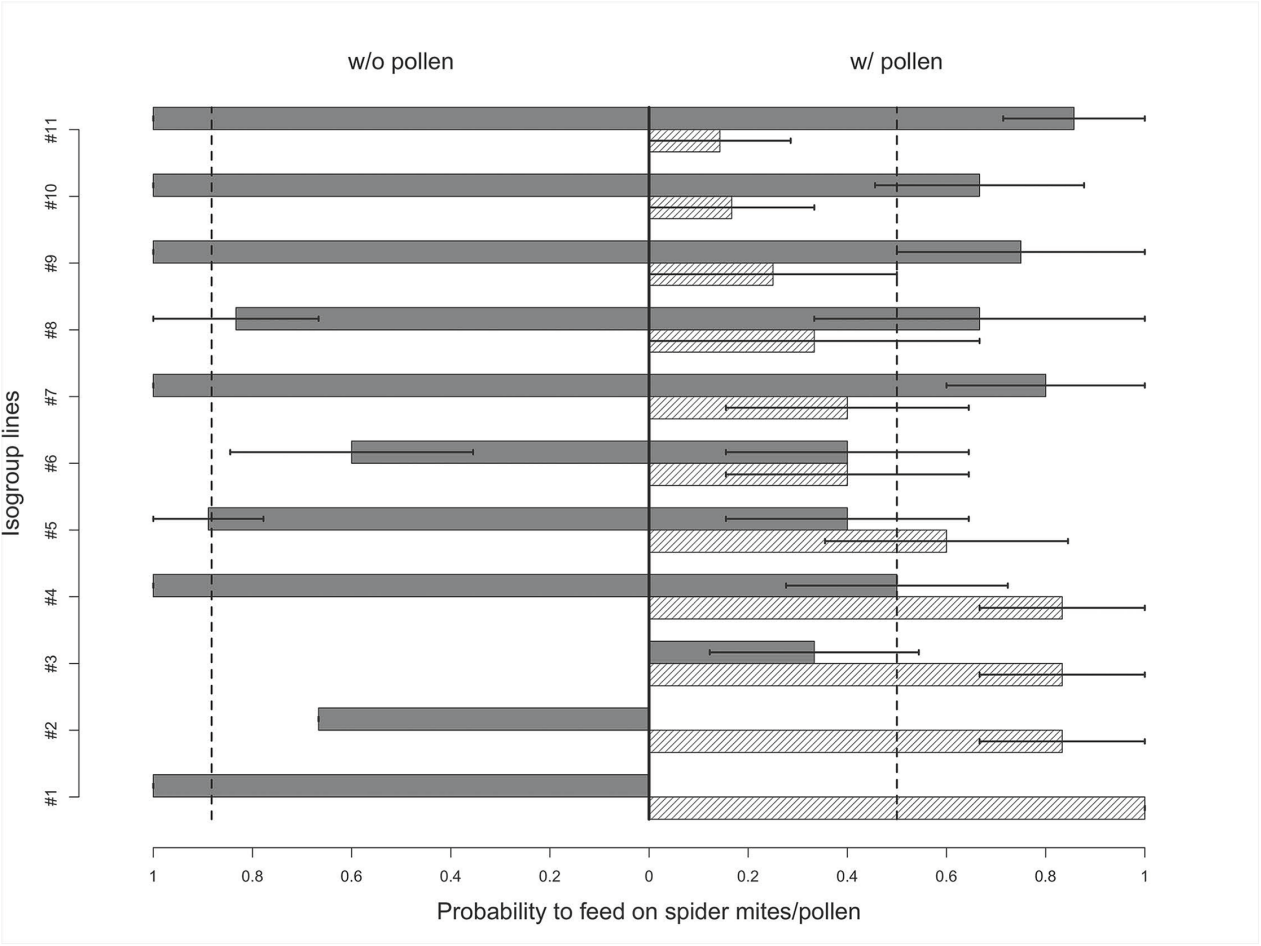
Probability of nymphal feeding on spider mites (dark bars) or pollen (light dashed bars) for 11 mullein bug isogroup lines, during a 15-min test, in two treatments without (left side) or with pollen (right side). Dashed black lines represent a population's mean probability of feeding on spider mites in both treatments.

In the without-pollen treatment, 88.3 ± 32.5% of the mullein bug nymphs (n = 51) fed on spider mites. In contrast, only 50.0 ± 50.4% of bugs fed on spider mites in the pollen treatment tests (n = 51). This difference was statistically significant (LRT = 13.25, df = 1, p < 0.0003). Development stage did not have any effect on zoophagy (LRT = 0.007, df = 2, p = 0.99), nor did generation (LRT = 0.90, df = 1, p = 0.34). The selected model included only line ID a random effect; lines differed significantly in their zoophagy (Fig 1, Table 1). Heritability (*h*^2^) of zoophagy (in both treatments) was estimated to be 0.28. Lines did not show any significant differences in their reaction norms in response to pollen availability, and zoophagy did not change across generations.

In the pollen treatment, 51.8 ± 50.4% of mullein bug nymphs fed on pollen. Neither developmental stages (LRT = 3.04, df = 2, p = 0.22) nor generation had significant effects on phytophagy (LRT = 0.01, df = 1, p = 0.91). The best-fitted model included line ID as random effect, indicating that lines showed a difference in phytophagy (Fig 1). Estimate of heritability (*h*^2^) of phytophagy was 0.20, after controlling for development stage and generation. The number of generations between the test and the foundation of the line had no significant effect on phytophagy.

**Fig 2:**
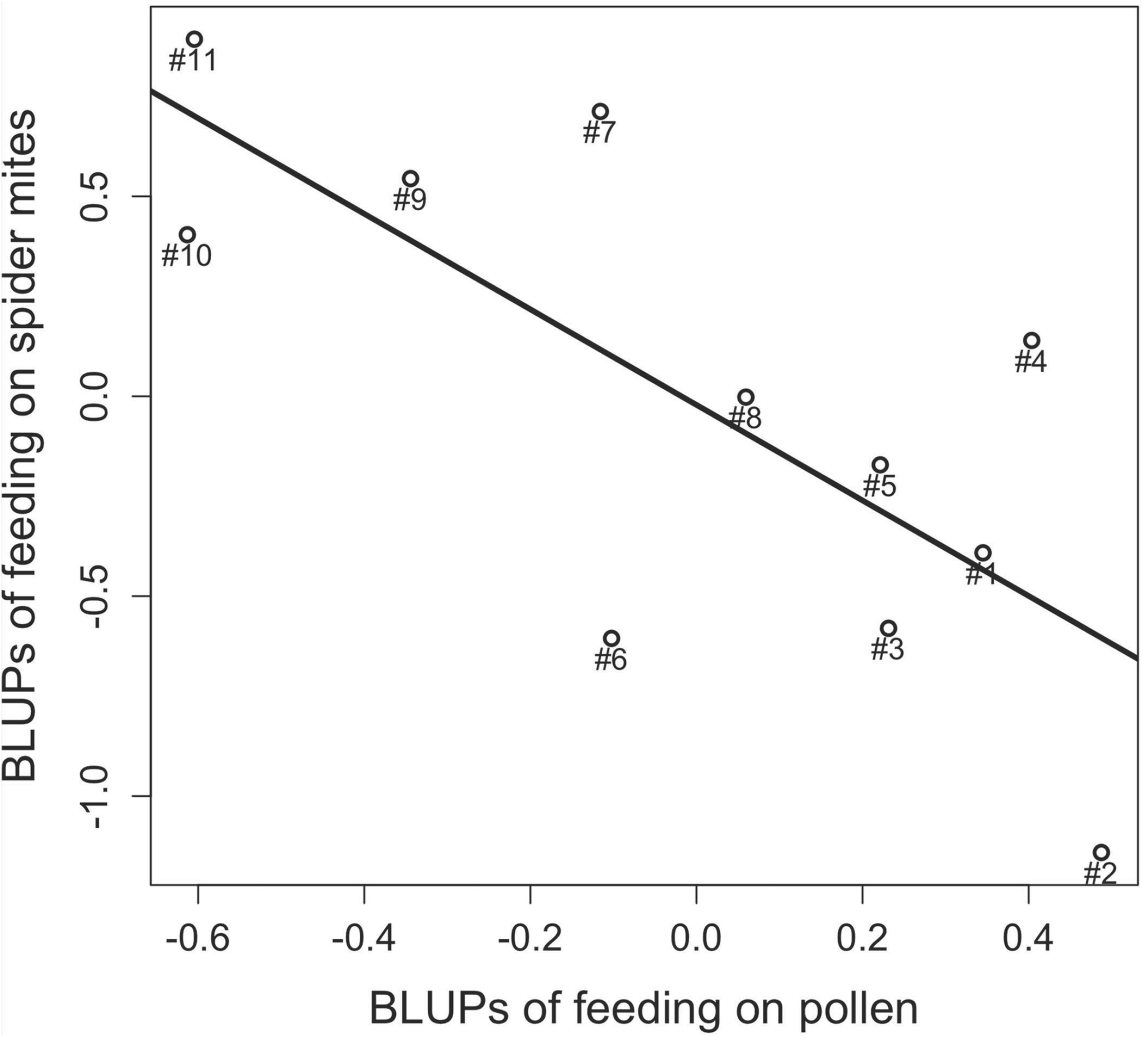
Correlation between the probability of feeding on spider mites and the probability of feeding on pollen for 11 mullein bug isogroup lines.

Isogroup-line zoophagy was significantly and negatively correlated with phytophagy (rho = -0.85; p = 0.002) (Fig 2).

**Fig 3:**
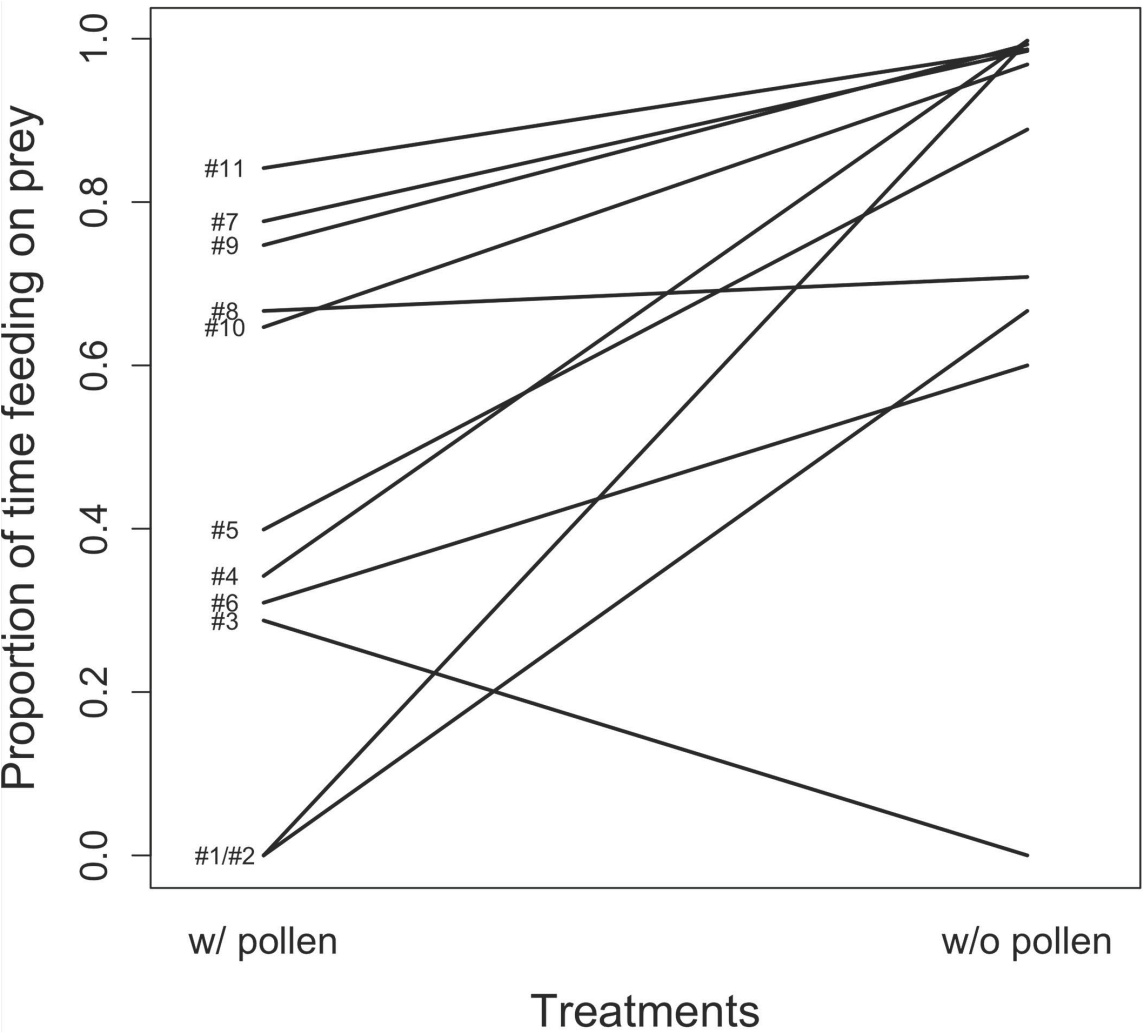
Effect of pollen availability on the proportion of time spent feeding on prey over the total time spent feeding, for mullein bug nymphs from 11 isogroup lines, during a 15 min. test. Numbers refer to isogroup lines in Fig 1.

In the without-pollen treatment, bugs spent 86.2 ± 33.5% of their total feeding time (all resources included) eating animal resources (i.e. two-spotted spider mite adults, nymphs and eggs; Fig 3). This proportion was 46.4 ± 48.2% when nymphs had access to pollen, which is not statistically significant (LRT = 2.77, df = 1, p = 0.10). The N3 to N5 nymphal stages varied in the proportion of time devoted to animal resources over time spent feeding on all the resources (LRT = 14.02, df = 2, p = 0.0009). There was no effect of generation on the proportion of time nymphs spent feeding on animal resources (LRT = 0.006, df = 1, p = 0.94). The model with the lowest AIC included line ID as random intercept and both generation and treatment as random slopes, as well as the correlation among these parameters (Table 1). Lines varied significantly in the proportion of time spent feeding on animal resources, and in their reaction norm in response to pollen availability (Fig 3). We found a heritability of 0.16 for the proportion of time spent feeding on prey when pollen was not available and of 0.68 when pollen was available. The negative correlation (r = -0.91) between the proportion of time spent feeding on prey in either treatment with or without pollen indicates that lines that mainly fed on pollen switched to prey when pollen was not available. The number of generations between the test and the foundation of the line had a significant effect on time spent feeding on prey. Variation in the proportion of animal resources across generations varied among lines (r = -0.25), indicating that genetic drift contributed to changes within lines but did not increase differences among lines.

## Discussion

Diet specialization results from the fact that individuals often exploit different subsets of the resources available to the population [3]. Such specialization can be caused by genetic variation in the ability to detect, capture, handle or digest different resources or in the response to changes in ecological conditions [7]. In zoophytophagous predators, diet specialization would be defined by among-individual differences in the proportions of animal and plant resources consumed and in changes in diet in response to changes in the relative abundance of each type of resources. Our results show that in the zoophytophagous mullein bug, individuals did not behave as generalists eating any type of resources as a proportion of their relative abundance, but there are genetic differences in the relative proportion of different types of resources eaten. In our study, we confirmed the hypotheses that lines differ in their trophic switching from prey to plant resources as a function of the absence or presence of pollen, revealing the existence of zoophytophagous and phytozoophagous lines. Furthermore, lines varied in their zoophagy regardless of whether or not they had access to pollen, although among-line differences in the level of zoophagy and thus heritability of diet specialization increased when pollen was provided as an additional resource. The absence of pollen compels phytozoophagous lines (that prefer pollen over prey) to switch to a diet with a high proportion of animal resources. Therefore, pollen was not replaced by other plant material (i.e. leaves). Thus, foraging behaviour in mullein bugs depends both on diet specialization and on the availability of spider mites and pollen as alternative resources.

When one of the main resources is absent (i.e. pollen) the differences between zoophytophagous and phytozoophagous strategies were reduced. In our tests the absence of pollen led to an increase in predation on spider mites, and as heritability of diet specialization decreased, individuals behaved more as generalists. In the case of zoophytophagous predators, the differences among individuals in voracity on a given resource would increase considerably when individuals have the choice between several resources. The genetic variation in the response to the availability of pollen we observed in mullein bug strongly supports this hypothesis. Consequently, the genetic diversity and the relative frequency of each type of foraging strategy within a population of zoophytophagous predators would be significant in the ecological and economical roles (i.e. benefits or damages to a crop that can result from zoophytophagous predators feeding behaviour and, hence, entail economic consequences in an agroecosystem) that these insects play in agro-ecosystem.

Zoophytophagous predators vary in their status depending on the region. For instance, the mullein bug is considered as a pest in apple and pear orchards in British Columbia, Nova Scotia, and New York State, but is reported as potentially beneficial in Québec, or England [12, 26]. Consequently, some authors have referred to mullein bugs as a zoophytophagous predators [27–29, 15], whereas others have qualified them as phytozoophagous [30, 31]. Our results showing genetically-based individual diet specialization are consistent with the hypothesis that mullein bug populations can vary in the relative proportion of zoophytophagous *vs* phytozoophagous genotypes. As a consequence, one may wonder what particular type of ecological or anthropic conditions drives the overall differentiation in the diet of different mullein bug populations. Pesticides can affect zoophytophagous predators directly by lowering their survival and reproduction, including plant-incorporated pest resistance [32–34], or indirectly by decreasing prey abundance [27]. Acaricides or systemic insecticides may affect zoophytophagous genotypes to a higher extent than phytozoophagous ones. First generation female mullein bugs lay their eggs either on herbaceous plants (e.g. mullein plants *Verbascum* spp.) or apple trees [12]. Zoophytophagous lines may choose apple tree hosts because spider mites may be abundant during summer (but pollen is not available). Nymphs emerging on apple trees during the mid-summer (i.e. July) could be affected to a higher extent than nymphs emerging on herbaceous plants by the chemical treatments used to control pests in orchards. Considering the relatively high heritability of the response to pollen availability (*h*^2^ = 0.68), such a process could rapidly become undesirable, as strong unintentional selection pressures favouring phytozoophagous genotypes may permanently increase the cost of sheltering more phytozoophagous bugs in the agro-ecosystem.

Genetic differences are usually ignored in most biological control studies, and the general practice is to assume ecologically equivalent and interchangeable individuals. However, ecological processes such as predator-prey interactions and dynamics depend on individual-level ecological interactions that can substantially vary following inter-individual variation [3, 8, 10, 35]. Nachappa et al. [36] observed that genetic variation in prey consumption, conversion efficiency and dispersal in predatory mites *Phytoseiulus persimilis* (Athias-Henriot) (Acarina: Phytoseiidae) affect predator-prey interactions, long-term population dynamics as well as efficiency in the biological control of two-spotted spider mites. In the zoophytophagous mullein bug, differences in zoophagy between lines might generate various levels of benefits in agro-ecosystems. The individual-based and genotypic-based approaches shed light on the ecological and economic roles of omnivorous predators. Our results on mullein bugs can be extended to other zoophytophagous mirids used in agronomy. Several zoophytophagous mirids such as *Macrolophus pygmaeus* (Rambur) (previously *M*. *caliginosus*), *Nesiodiocoris tenuis* (Reuter), *Dicyphus tamaninii* (Wagner), and *Dicyphus hesperus* (Knight) (Hemiptera: Miridae), are efficient predators of white flies *Bemisia tabaci* (Gennadius) (Hemiptera: Aleyrodidae) and tomato borer *Tuta absoluta* (Meyrick) (Lepidoptera: Gelechiidae) in tomato fields and greenhouses [37–41]. All of these predators can damage crops by puncturing fruits and/or vegetative parts [2, 42]. Just as the status of mullein bugs can vary drastically between populations, the status of several zoophytophagous mirids in tomato crops is highly controversial as their efficiency and propensity to damage plants and more specifically fruits, varied considerably across studies [43]. A study of the genetic variations underlying foraging behaviour in mirids could help improve our understanding of how population differences affect feeding habits in this ecologically and economically important hemipteran family.

### Ethics statement

Field-collected insects were obtained from either private land or public areas. In the case of private land, we had the agreement of owners. No specific permissions were required to collect the mullein bugs in public areas in accordance with Canadian laws. The field studies did not involve endangered or protected species.
